# Diphtheritic Conjunctivitis

**Published:** 1862-04

**Authors:** E. L. Holmes

**Affiliations:** Chicago


CHICAGO
MEDICAL JOURNAL.

VOL. V.] APRIL, 1862. [No. 4.

### ORIGINAL COMMUNICATIONS.

### DIPHTHERITIC CONJUNCTIVITIS.

By E. L. HOLMES, M. D., of Chicago.

There is perhaps no disease to which the attention of the profession and of the public has been directed for the past few years so frequently as to diphtheria.

Especial interest has been awakened in this country from the fact that so long a period had elapsed since the last appearance of the disease, that physicians were almost wholly without experience in its treatment at the commencement of the present epidemic; this interest has been increased by the fatality of the disease which has too often baffled the most scientific skill and carried sadness and grief into thousands of families. The disease is remarkable, we believe, not only for the duration of the epidemic, but also for the length of time which may elapse from one epidemic to another.

The medical journals throughout the country have published numerous articles upon the subject, and have reported cases illustrating the peculiarities of the affection; but we are not aware that much has been written in regard to diphtheritic inflammation of the conjunctiva. This is probably to be explained by the fact that this disease of the eye is very rare in this country. We believe the same is true of diphtheria affecting other portions of the mucous membrane than those of the mouth and throat, leech bites and surfaces of the body denuded by burns and blisters, as reported not unfrequently by European observers. In an aggregate number of more than ten thousand cases of diseases of the eye, tabulated in several annual Reports of the New York Eye Infirmary, there is not a case of diphtheritic conjunctivitis. In eleven hundred cases of ophthalmic disease which we have treated in Chicago, we have met with true diphtheritic membrane in only one instance. Upon enquiry also among our medical friends with extensive practice in Chicago and different portions of the State we have heard of only one patient affected with this disease. We have had an opportunity of examining but few of our Medical Journals, and are consequently ignorant of cases which may have been reported in various parts of our country. Prof. Clark, in his lectures on Diphtheria in the American Medical Times, vol. ii. 1861, alludes to several cases which fell under the observation of Dr. Rives at the Children's Hospital on Randall's Island. He also published in one of these lectures the history of one case observed by Dr. H. D. Noyes of the New York Eye Infirmary. As the disease is seldom mentioned in the London ophthalmic hospital Reports, it may be inferred that it is also rare in England. On the Continent of Europe, however, the disease is quite common.

The three most reliable papers upon Diphtheritic conjunctivitis with which we are acquainted are one by V. Graefe, published in the first volume of the archiv fur Opthalmologie, one by Jacobson in the sixth volume of the same work, and another by Dr. Lewinski in the Annalles d'omlistique 1861.

The article by V. Graefe is said to be the most scientific and comprehensive that can be found in any language. It contains a thorough investigation of nearly every thing pertaining to the disease, including an accurate description of the peculiarities which distinguish it from other forms of acute inflammation of the conjunctiva, in reference to diagnosis and treatment. The article has been translated and published in the London Medical Review. We would recommend these papers to the consideration of all those interested in this subject.

A typical case of diphtheritic conjunctivitis has certain peculiarities, which characterize it from muco-purulent (blenorrhœa) inflammation. "In the latter, the conjunctiva is spongy, œdematous, filled with a fluid exudation, while in diphtheria it is unyielding and filled with a firm fibrinous exudation; in the one the lid will be found soft, puffy and easily turned, but in the latter stiff and less movable. The conjunctiva in Blenorrhœa is supplied with an unnatural amount of blood and the circulation is free in the greater part of its vessels. The diphtheritic conjunctiva on the other hand is in a great measure deprived of its supply of blood in consequence of the great firmness of the exudation, causing an undue pressure upon the vessels."

The discharge from the mucous membrane in Blenorrhœa is a homogeneous pus, while in Diphtheria it is thin, of a dirty gray color and more irritating or even corrosive in character.

The heat is much higher in the diphtheritic than in the other form of inflammation. In the latter disease, wet (cold) compresses require changing every two or three minutes; in the former they become much more rapidly heated, requiring change every twenty-five or thirty seconds.

The pain is much greater in the former than the latter.

The swelling of the conjunctiva, independent of the submucous membrane is greater in the diphtheritic than in the purulent inflammation, as may be seen by examining a section of the conjunctiva in each disease after scarification.

The following is the usual course of the disease: An eye, previously to all appearance, healthy, or more frequently an eye, which has been affected with inflammation is suddenly attacked with sensations of pain, heat and swelling accompanied with more or less increased secretion of tears. The swelling of the upper lid soon becomes so great that all its folds are obliterated. Upon turning the lids the conjunctiva instead of presenting the usual red, rough, vascular, appearance peculiar to ordinary severe inflammation is smooth, yellowish and glistening and an inexperienced observer, for this reason would imagine that the affection was less serious than the other. And yet, in a vast majority of instances suitable treatment will relieve the latter disease, while the former proves in a large number of cases even with the best treatment one of the most dangerous, to which the eye is liable. The conjunctiva of the lids and sometimes of the globe becomes infiltrated with a firm organizable exudation, which in the most severe cases almost wholly destroys the circulation in this tissue. The exudation is also thrown out upon the surface of the conjunctiva forming a layer varying from a half a line to a whole line in thickness. The microscopical appearances of this membrane are well described in the first lecture of Prof. Clark above mentioned. This process of exudation, which takes place during the first stage of the disease in mild cases lasts but two or three days—in severer cases it contains six or eight or even ten days. At the termination of the first stage the membrane commences to exfoliate—occasionally in a single large layer of the form of a lid, but usually in small fragments, leaving a red, roughened, bleeding surface, which is the commencement of what may be called the second or blenorrhoeal stage, characterized by a mucopurulent discharge. Not unfrequently after the exfoliation, the membrane is renewed and again cast off—this process being in some cases repeated several times. The duration of this stage varies in length even more than the first. The third stage, when the disease is not arrested in its course, is distinguished by atrophy of the conjunctiva and even of the lid, in consequence of insufficient nutrition caused by obstruction of the arterial and venous circulation. The absorption of the conjunctiva is sometimes so great that the edges of the lids are bound quite firmly to the globe.

The prognosis as regards vision depends almost entirely upon the condition of the cornea. In consequence of the great derangement in the circulation and nutrition of the cornea and its adjoining tissues, the cornea very often, early in the course of the disease, presents a cloudy appearance over a greater or less extent of its surface. In twelve or twenty-four hours, the epithelium is removed and ulceration commences which often disorganizes a large portion of this important membrane. The prognosis is generally less favorable for adults than for children. The amount of exudation, and chemosis, the rigidity of the lids and the degree of ulceration of the corner are symptoms of the greatest importance as regards the ultimate result.

Of the true causes of diphtheritic conjunctivitis little can be said. It is undoubtedly a constitutional disease, the affection of the eyes being symptomatic. While mucopurulent conjunctivitis seems frequently to attack individuals in perfect health, as readily as those suffering from other diseases, this form of inflammation is almost invariably found in patients with health more or less impaired. Nearly a fifth of a series of cases in children (forty in number) observed by V. Graefe, bore undoubted signs of congenital syphilis. It is stated that the disease in this country has generally attacked patients affected with measles or scarlet fever.

The disease in Europe usually appears as an epidemic, as is shown by the observations of those who have had most experience. V. Graefe states that in his clinic, in which four or five thousand patients are annually treated with diseases of the eye, the affection disappears for months and again returns, following about the same laws in this respect as diphtheritic inflammation of the throat.

In reference to the period of life most subject to this disease, Graefe's cases show that one in fifty patients are attacked during the second half of the first year. Nearly one-half of his patients were between the ages of eighteen and thirty-six months. As the age increases beyond eight years, the disease becomes quite rare. The disease can be communicated to a healthy eye by direct contact of the abnormal discharges. This almost invariably produces an inflammation of the most violent form, which, however, is not necessarily diphtheritic, but in many instances simply muco-purulent.

It would appear from the reports of cases, which we have been able to consult, that no absolutely satisfactory mode of treatment has as yet been discovered. Oculists of experience do not agree in reference to the use of calomel, caustic applications and local depletion, either by leeches or scarifications. The best authorities, however, have abandoned the use of caustics in marked cases of this disease during the first stage. Graefe recommended the free application of leeches to the side of the nose near the inner angle of the eye, and especially of compresses dipped in water. These should be changed day and night every few minutes. Particular care should be taken to remove the disharges from under the lids. Graefe recommends milk as a simple and soothing application for cleansing inflamed conjunctiva. The same author speaks decidedly in favor of full doses of calomel and especially of the endermic use of Ung. Hyd. until marked constitutional effects are produced. He states that the exudation loses its firmness and the disease often assumes the form of simple muco-purulent inflammation after their use. Patients should be confined to a liquid diet. Under this treatment more than two-thirds of Graefe's cases recovered, while scarcely a single favorable result attended less appropriate treatment.

In severe cases of diphtheritic inflammation of the eye, where not only the substance of the conjunctiva is infiltrated with a firmly organized lymph, but its surface covered with a tenacious membrane, we are certain no caustic application will arrest the disease. Incisions must be carried to great depth in order to produce depletion. It is doubtful whether much advantage can be accomplished in this way, since the incisions are soon closed with lymph, preventing much escape of blood. In the second stage of the disease, after the mem brane has been thrown off and the surface of the conjunctiva presents the appearances of ordinary purulent inflammation, caustics and astringents may be used with benefit.

Although we believe the disease in its most violent form seldom leaves the cornea clear or the lids in their natural form, it is stated that sporadic cases are much less dangerous. It should be remembered that occasionally in ordinary inflammation of the conjunctiva, small patches of diphtheritic membrane will be formed upon the diseased surface. Such cases are usually quite mild. With these patches, however, should not be confounded circumscribed elevations, which are somewhat similar in appearance to them, but which are simple thickened mucous adhering to the surface and are not to be regarded as a serious symptom.

